# AMYCNE: Confident copy number assessment using whole genome sequencing data

**DOI:** 10.1371/journal.pone.0189710

**Published:** 2018-03-26

**Authors:** Jesper Eisfeldt, Daniel Nilsson, Johanna C. Andersson-Assarsson, Anna Lindstrand

**Affiliations:** 1 Department of Molecular Medicine and Surgery, and Center for Molecular Medicine, Karolinska Institutet, Stockholm, Sweden; 2 Science for Life Laboratory, Karolinska Institutet Science Park, Solna, Sweden; 3 Department of Clinical Genetics, Karolinska University Hospital, Stockholm, Sweden; 4 Department of Molecular and Clinical Medicine, Sahlgrenska Academy, University of Gothenburg, Gothenburg, Sweden; Hospital Authority, CHINA

## Abstract

Copy number variations (CNVs) within the human genome have been linked to a diversity of inherited diseases and phenotypic traits. The currently used methodology to measure copy numbers has limited resolution and/or precision, especially for regions with more than 4 copies. Whole genome sequencing (WGS) offers an alternative data source to allow for the detection and characterization of the copy number across different genomic regions in a single experiment. A plethora of tools have been developed to utilize WGS data for CNV detection. None of these tools are designed specifically to accurately estimate copy numbers of complex regions in a small cohort or clinical setting. Herein, we present AMYCNE (automatic modeling functionality for copy number estimation), a CNV analysis tool using WGS data. AMYCNE is multifunctional and performs copy number estimation of complex regions, annotation of VCF files, and CNV detection on individual samples. The performance of AMYCNE was evaluated using *AMY1A* ddPCR measurements from 86 unrelated individuals. In addition, we validated the accuracy of AMYCNE copy number predictions on two additional genes (*FCGR3A* and *FCGR3B)* using datasets available through the 1000 genomes consortium. Finally, we simulated levels of mosaic loss and gain of chromosome X and used this dataset for benchmarking AMYCNE. The results show a high concordance between AMYCNE and ddPCR, validating the use of AMYCNE to measure tandem *AMY1* repeats with high accuracy. This opens up new possibilities for the use of WGS for accurate copy number determination of other complex regions in the genome in small cohorts or single individuals.

## Introduction

The human genome is rich in copy number variable regions. It is estimated that up to 10% of the human genome is affected by CNVs [1]. Thus, it is not surprising that CNVs contribute greatly to genetic diversity and affect the phenotypic traits of an individual [2]. There is a large variety of CNVs, comprising events leading to both gain and loss of genetic material. The variation in genetic copy number may also cause a dosage effect of genes affected by the CNV [3].

The *AMY1* locus is an example of such a CNV. *AMY1* encodes the human salivary α-amylase, which is located in a multi-allelic copy number variable region on chromosome 1 [4]. Each *AMY1* copy is functional and affects the protein levels of salivary α-amylase [4,5].

The role of salivary α-amylase is to hydrolyze complex carbohydrates such as starch into simple carbohydrates (maltose and glucose) [4]. Furthermore, the number of *AMY1* copies significantly affects the energy uptake of an individual. Several recent studies show an inverse correlation between BMI and *AMY1* copy number [5].

The current standard method to assess the exact copy number of a genomic region is targeted ddPCR [6]. In a ddPCR reaction thousands of PCRs are performed simultaneously inside small droplets [7]. Due to the high number of reactions performed, a ddPCR experiment is highly accurate. However, one of the main drawbacks of ddPCR is that the number of measured regions per experiment is limited. In contrast, whole genome sequencing (WGS) may be utilized for CNV detection [8] and copy number estimation [9] across the entire genome based on only one experiment. Few tools are available for the study of CNVs in complex genomic regions that are represented as multiple separate loci in the reference (S1 Fig).

Here, we present AMYCNE (automatic modeling functionality for copy number estimation), a tool that utilizes WGS data to compute the copy number of specific regions in the genome. These genomic regions, known as target regions are selected with a simple and powerful syntax, which allows for the addition as well as averaging of the copy number of genomic regions. The AMYCNE package may also be used to estimate and annotate the copy number of regions using structural variant VCF files. Moreover, AMYCNE contains a module used for the detection of large CNVs and chromosomal aneuploidies. Large and complex CNVs are generally difficult to detect using split read or discordant pair information exclusively [10]. The AMYCNE package may therefore be used to complement such structural variant callers. To our knowledge, the AMYCNE package allows for the most accurate and efficient copy number analysis of complex CNV regions.

## Methods

### Algorithms

#### Copy number estimation

AMYCNE requires three input files to run; one file describing the target regions, and two bed files describing the coverage and GC-content of non-overlapping bins covering the entire genome. The first three fields of the bed files describe chromosome, start position and end position of each bin. The fourth field describes the GC content or coverage of the specific bin, *bin*_*i*_. The coverage bed file may contain an optional fifth column, describing the average mapping quality of reads within that bin.

AMYCNE contains a module for computing the GC content of bins across any genome stored in a FASTA file. This module will filter genomic regions that contain a large amount of undetermined bases N by setting their GC content to -1. The default threshold of this filter is to flag regions having an N content 20% or higher. Moreover, low complexity bins are set to -1, and will be excluded from the analysis. The complexity of a bin is found by computing the frequency of the most frequent 3*mer*, 3*mer*_*max*_ within the sequence of that bin, such that

$$f_{{3mer}_{max}}=\frac{{3-mer}_{max}}{L-2},$$

where *L* is the bin size. If the frequency, $f_{{3mer}_{max}}$ is higher than 0.2, the region is deemed repetitive. In a bin were the complexity is maximal, each possible 3mer should be equally frequent, so $f_{{3mer}_{max}}$ will be equal to $\frac{1}{64}$.

To estimate the copy number in one or more user set regions, AMYCNE performs a three-step procedure. The target region *R* may be selected in three ways, either by using a structural variant VCF file, a BED file, or by using an AMYCNE region file. This is described in more detail in the target regions section.

**The initiation step:** Before estimating the copy number of the target region, the initiation step is performed. The purpose of the initiation step is to read and store the input data. Each bin, *bin*_*i*_ is defined as a 5-tuple describing the position of the bin, the GC content, *GC*_*i*_ and coverage, *C*_*i*_, such that

$${bin}_{i}=({chr}_{i},{start}_{i},{end}_{i},{GC}_{i},C_{i}),\forall i\in 1,2,3,\ldots ,n,$$

where *chr*_*i*_, *start*_*i*_, *end*_*i*_ describes the chromosome, start, and end position of *bin*_*i*_, and n is the total number of bins.

**The reference initiation step:** Once each bin is populated, the initiation step is initiated. This step aims to create an estimate of the coverage of a copy number neutral bin for every distinct GC content value, k. This reference value, ${GC}_{k}^{ref}$, is found by computing the average value of each *C*_*i*_ whose *GC*_*i*_ = *k*.

Given that |*C*_*i*_: *GC*_*i*_ = *k*| is large, ${GC}_{k}^{ref}$ will be close to the expected coverage value of a bin having GC content *k*. To decrease the risk of using abnormal reference values, two thresholds exist, *S*_*cutoff*_ and *C*_*cutoff*_. Both of these thresholds must be satisfied in order to use a certain reference value, and any ${GC}_{k}^{ref}$ that do not satisfy the threshold values will be discarded. The threshold S_*cutoff*_ is used to remove any ${GC}_{k}^{ref}$ constructed from an inadequate number of bins (default 50). The purpose of *C*_*cutoff*_ is to remove any ${GC}_{k}^{ref}$ having abnormal coverage. As a default value, the average coverage of the bins of *GCkref* must be between 10 or 100 or it will be discarded. The user may change *C*_*cutoff*_ or *S*_*cutoff*_ to any suitable number. To increase the accuracy of AMYCNE, the sex chromosomes are excluded from the construction of the reference. If the coverage BED file contains mapping quality information for each bin, bins having a mapping quality below a user set value will be rejected from the reference construction step.

**The copy number estimation phase:** Finally, in the copy number estimation phase, the copy number of each non-excluded bin, *bin*_*r*_, within the target region, *R*, is estimated. Bins are excluded if they have a GC content set to -1 or if their reference *GCkref* failed the quality checks implemented in the reference initiation step. The copy number, *CN*_*r*_ of the non-excluded bins is estimated through this formula:

$${CN}_{r}=P*\frac{C_{r}}{{GC}_{k}^{ref}},$$

where *P* is the ploidy of the organism. By computing *CN*_*r*_ for each bin within *R*, a copy number distribution will be obtained. The copy number of *R*, ${CN}_{r}^{tot}$ is found by computing the average value of every *CN*_*r*_. The precision of the copy number estimation is found by computing a 95% a confidence interval, *Ci*, of the normal distribution of *CN*_*r*_.

$$Ci={CN}_{r}^{tot}\pm 1.96\frac{\sigma }{\sqrt{n}},$$

where *σ* is the standard deviation of *CN*_*r*_ and *n* is the total number of non-excluded bins within *R*. Upon finishing the copy number estimation phase, the values ${CN}_{r}^{tot}$, the rounded ${CN}_{r}^{tot}$, *n*, *Ci*, and the fraction of non-excluded bins within *R* are reported to the user.

### Selection of target regions

Three separate types of input files may be used to define the target region *R*, either a structural variant VCF file, a BED file, or an AMYCNE region file. If the target region is defined in a structural variant VCF file, AMYCNE will compute the copy number across each intrachromosomal variant, and add the statistics described in the previous section as tags within the VCF INFO field. If the target region is specified using a BED file, the copy number of the AMYCNE statistics will be added as new columns to the input BED file. When the target region is specified using an AMYCNE region file, the statistics reported by AMYCNE will be presented as a tab separated table printed to standard output.

**The AMYCNE region file:** In an AMYCNE region file, each region and operation is described as only one line. The average and sum operations are supported. These operations are applied on any number of sub regions specified on that line. Thus, the copy number of multiple regions may be summed or averaged into one copy number estimate. The AMYCNE region file is given in the following format:

sum(chrA:start-end|chrB:start-end[|…])

avg(chrA:start-end|chrB:start-end[|…]),

where sum means that the copy number of the regions on chrA and chrB will be summed, while avg means that the average copy number of the regions will be reported. If sum is chosen, each *CN*_*r*_ will be multiplied by the number of sub-regions. There is no limit on the number of lines that may be added to one single AMYCNE region file, nor is there any limit on the number of sub-regions per operation.

The purpose of the summing operation is to add the copy number of one genomic region that is represented as multiple distinct, but similar regions in the reference. One such example is the highly copy number variable *AMY1* locus on chromosome 1p21.1 that is in fact represented by three genes, *AMY1A*, *AMY1B*, and *AMY1C* [11]. To obtain an accurate estimate of the true copy numbers, all regions must be accounted for in the copy number estimation step.

The averaging operation is used to skip problematic regions that may degrade the copy number estimation, such as regions of low mappability. When choosing the averaging operation, the bins of all target sub-regions are used to estimate ${CN}_{r}^{tot}$. Thus ${CN}_{r}^{tot}$ will become the average copy number of all sub-regions.

### ddPCR

*AMY1* copy number was determined using the QX200 droplet digital PCR system (Bio-Rad, Hercules, CA). Each reaction consisted of 11μl ddPCR Supermix for Probes (no dUTP, Bio-Rad), 0.8 μl FAM-labeled Copy Number Detection Assay for *AMY1* (900nM primers and 250 nM probes, Assay ID: dHsaCP1000594, Bio-Rad), 0.8 μl HEX-labeled Copy Number Detection Assay for *AP3B1* (900nM primers and 250mM probes, Assay ID: dHsaCP2500348, Bio-Rad), 3U of HaeIII (New England Biolabs), 10-20ng of DNA and water to yield a final volume of 22 μl. A ViaFlo 384 pipetting robot (Integra) was used to mix the reactions thoroughly before droplet generation to ensure an even distribution of sample and reagents in each individual droplet. The plates were then heat-sealed and droplets were generated using an Automated Droplet Generator (Bio-Rad). After droplet generation, the sample plate was heat-sealed and PCR was performed in a C1000 Touch thermal cycler (Bio-Rad) with the following program: 95°C for 10 minutes followed by 40 cycles of 94°C for 30 seconds and 60 °C for 60 seconds with a ramp speed of 2°C/sec. Samples were then denaturated at 98°C for 10 minutes and kept at 4°C until reading, usually over-night. Droplets were read in a Droplet Reader (Bio-Rad). A negative control (water), as well as two to three control samples with known copy number, were included in each run. Initial data quality control and copy number determination was performed using QuantaSoft v1.7.4 (Bio-Rad).

### Benchmarking of AMYCNE

The performance of AMYCNE was tested in multiple settings. AMYCNEs capabilities of annotating CNVs were tested on the WGS data from the NA12878 sample [12]. Next, we assessed the accuracy of AMYCNE by estimating the *AMY1* copy number of 86 individual samples. These estimates were compared to ddPCR results, as well as two WGS analysis methods, namely the diploid copy number estimation (DCNE) of *AMY1* method [13] and CNVnator [8]. Additionally, we analyzed the copy numbers of *FCGR3A* and *FCGR3B* in data available from 164 individuals that had been whole genome sequenced through the 1000 genomes project and genotyped in a previous study [14]. Lastly, AMYCNE was tested on a simulated dataset representing 20 carriers of mosaic aneuploidies of chromosome X. The scripts and methods used to analyze these datasets are presented in the supplementary methods (S1 Text).

### Copy number estimation of *AMY1*

Assuming that ddPCR is more accurate than the WGS analysis methods, the WGS method whose results are the most similar to ddPCR will be the most accurate. The difference between the ddPCR measurements and the WGS copy number predictions was calculated for each WGS prediction method of each sample. The accuracy of the WGS prediction methods was then evaluated by computing the mean absolute error (MAE), standard deviation of the absolute error (SDAE), and accuracy. The accuracy is defined as the number of correct estimates divided by the total number of samples. Finally, the Wilcoxon signed-rank test was used to test if there was a significant difference between AMYCNE and the two other prediction methods. The Wilcoxon signed-rank test was employed by comparing AMYCNE to the other two WGS prediction methods, one at a time. The sign of each sample was determined by comparing the absolute error of AMYCNE to the absolute error of each separate WGS prediction method. The accuracy of AMYCNE was further tested by comparing the *AMY* genes copy number distributions of the 86 samples to the findings of recent studies [11,15].

### Copy number estimation of *FCGR3A* and *FCGR3B*

Copy number measurements of *FCGR3A* and *FCGR3B* was available for 164 individuals from the 1000 genomes cohort [14,16]. The low coverage whole genome data available through the 1000 genomes consortium from the 164 individuals was analyzed using AMYCNE and CNVnator and the results were compared to the truth set presented in [16], using the same procedure as presented for the *AMY1* locus. DCNE of AMY1 was not applied to this test since it is designed specifically for genotyping of *AMY1*.

### Copy number estimation of mosaic aneuploidies of chromosome X

The performance of AMYCNE was tested on a simulated WGS dataset consisting of 20 individuals carrying various mosaic aneuploidies of chromosome X. The simulation was done using Simseq (https://github.com/jstjohn/SimSeq) and the detailed method used is given in (S1 Text). The performance of AMYCNE was then compared to CNVnator as well as a simplistic method involving scaling of the coverage of chromosome X to the overall genomic coverage. The results of all three methods were evaluated in a similar fashion to the benchmarking on the *AMY1* locus.

### Annotation of DELLY deletion calls of the hapmap sample NA12878

NA12878 is a public sample sequenced through the platinum genomes project [12]. The deletions present in this sample were analyzed using DELLY, which is a variant caller that utilizes paired-end and split read information to call and classify variants [17]. A set of validated deletions has been found for the NA12878 sample [18]. This set of deletions was used to assess the precision and sensitivity of DELLY. The similarity of the called variants and the validated variants was determined based on reciprocal overlap. If the overlap between a called variant and a variant in the set of validated variants is higher than 0.6, the variants are deemed to be the same.

The AMYCNE annotation module was benchmarked by annotating the DELLY output files. These files were then filtered using a filter script. Thereafter, the precision and sensitivity of DELLY before and after filtering was compared. Since AMYCNE is aimed to estimate the copy number of large variants, all deletions smaller than 500 base pairs were removed from the evaluation.

### Preprocessing of the WGS data

A total of 86 individuals with available 30x paired-end WGS were analysed. These individuals were sequenced in a project aimed at validating WGS for clinical structural variant detection. The samples were sequenced at Science for Life Laboratory (Solna, Sweden) through the National Genomics Infrastructure (NGI). A PCR-free paired-end (2*150) library was used, the output data was preprocessed using the NGI best practices pipeline Piper, https://github.com/NationalGenomicsInfrastructure/piper. This pipeline performed an alignment to the hg19 reference genome using the Burrows-Wheeler Algorithm (BWA; MEM-algorithm, version 0.7.4-r385) [19] and resulted in 30X mean coverage of the samples (range 15-40X coverage). The coverage BED files were generated using TIDDIT (https://github.com/SciLifeLab/TIDDIT) with the bin size set to 100 bases. This study was approved by the regional ethics board in Stockholm.

## Results

### Copy number estimation of the *AMY1* locus

AMYCNE, CNVnator, and the diploid copy number estimation of *AMY1* method proposed by [11] was used to estimate the *AMY1* copy number of the 86 individuals as presented in the methods section. It was found that AMYCNE is most accurate method (Table 1). Utilizing the one-sided Wilcoxon signed-rank test, we found that the differences in accuracy between AMYCNE and the two other methods was significant (CNVnator P value = 0.0, DCNE of *AMY1* P value = 0.03).

**Table 1:** Comparison of AMYCNE, DCNE of *AMY1*, and CNVnator against the *AMY1* ddPCR measurements.

Copy number estimation of the *AMY1* Locus			
	AMYCNE	DCNE of *AMY1*	CNVnator
MAE	0.29	0.55	0.62
SDAE	0.9	1.23	0.94
Accuracy	0.86	0.74	0.58

MAE = mean average error; SDAE = standard deviation of the average error

**Fig 1:**
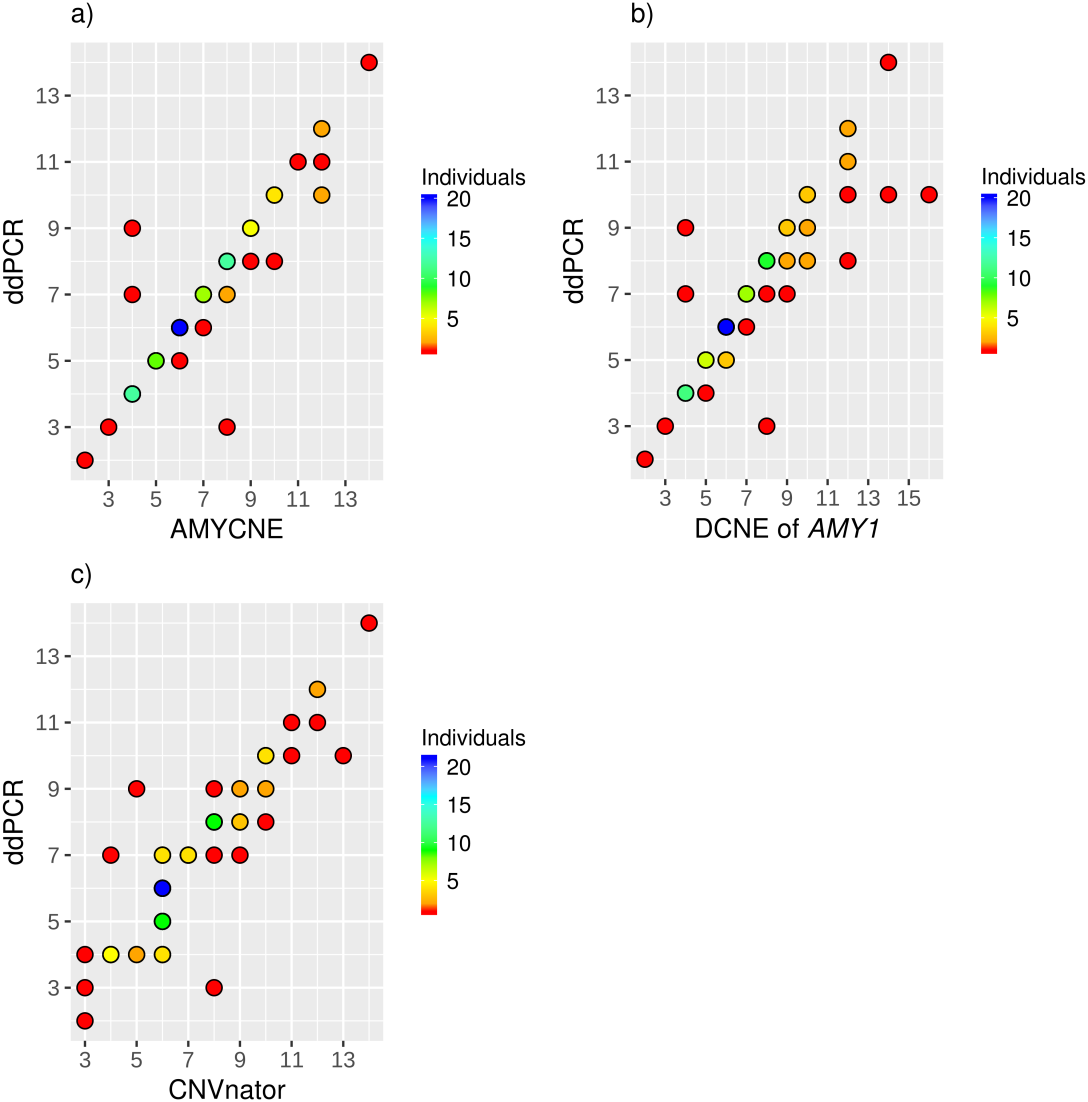
A comparison of three WGS copy number estimation methods. A comparison of the three WGS copy number estimation methods: (a) AMYCNE, (b) Diploid copy number estimation of *AMY1*, and (c) CNVnator. In all heat maps the number of samples per point is illustrated by color from red (low numbers) to blue (high numbers) as shown in the color key.

The DCNE of *AMY1* produce a larger spread across the entire interval of *AMY1* copy numbers than AMYCNE (Fig 1). The accuracy of the CNVnator is low at copy numbers less than 8. Instead, CNVnator tends to classify most copy numbers as either 3 or 6. Once the copy number reach 8 or higher, the performance of CNVnator is somewhere in between AMYCNE and DNCE of *AMY1* (Fig 1).

**Fig 2:**
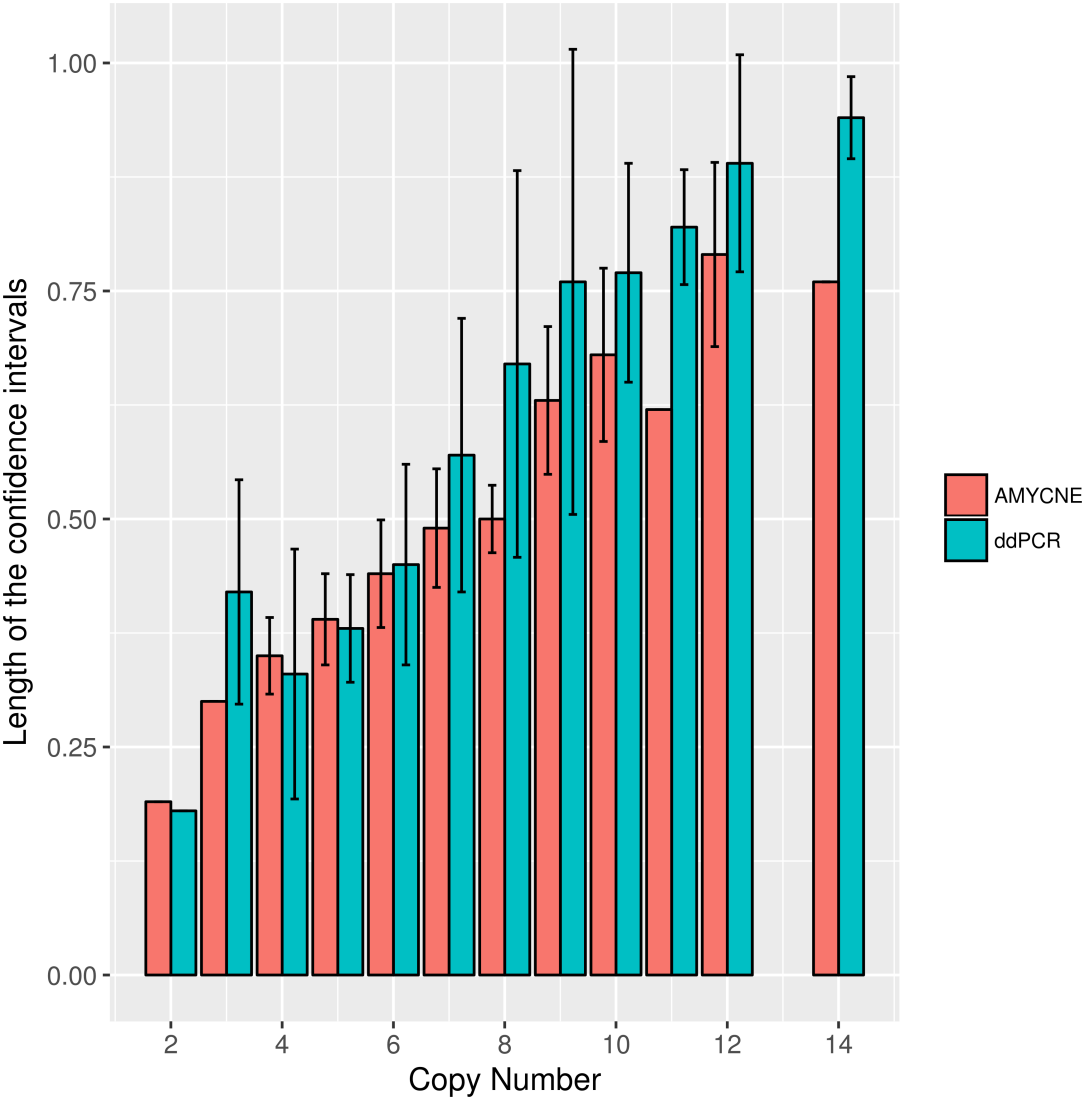
The precision of AMYCNE and ddPCR measurements. A comparison of the length of confidence intervals and copy number reported by AMYCNE and ddPCR in red and blue respectively.

The precision of the predictions made by ddPCR and AMYCNE is reported using 95% confidence intervals (CI). It was found that the Spearman correlation between CI length and copy number predictions of the ddPCR and AMYCNE measurements was 0.98 and 0.97, respectively. This implies that the precision of ddPCR and AMYCNE is getting lower as the copy number increases (Fig 2).

### Copy number distributions of *AMY1*, *AMY2A* and *AMY2B*

**Fig 3:**
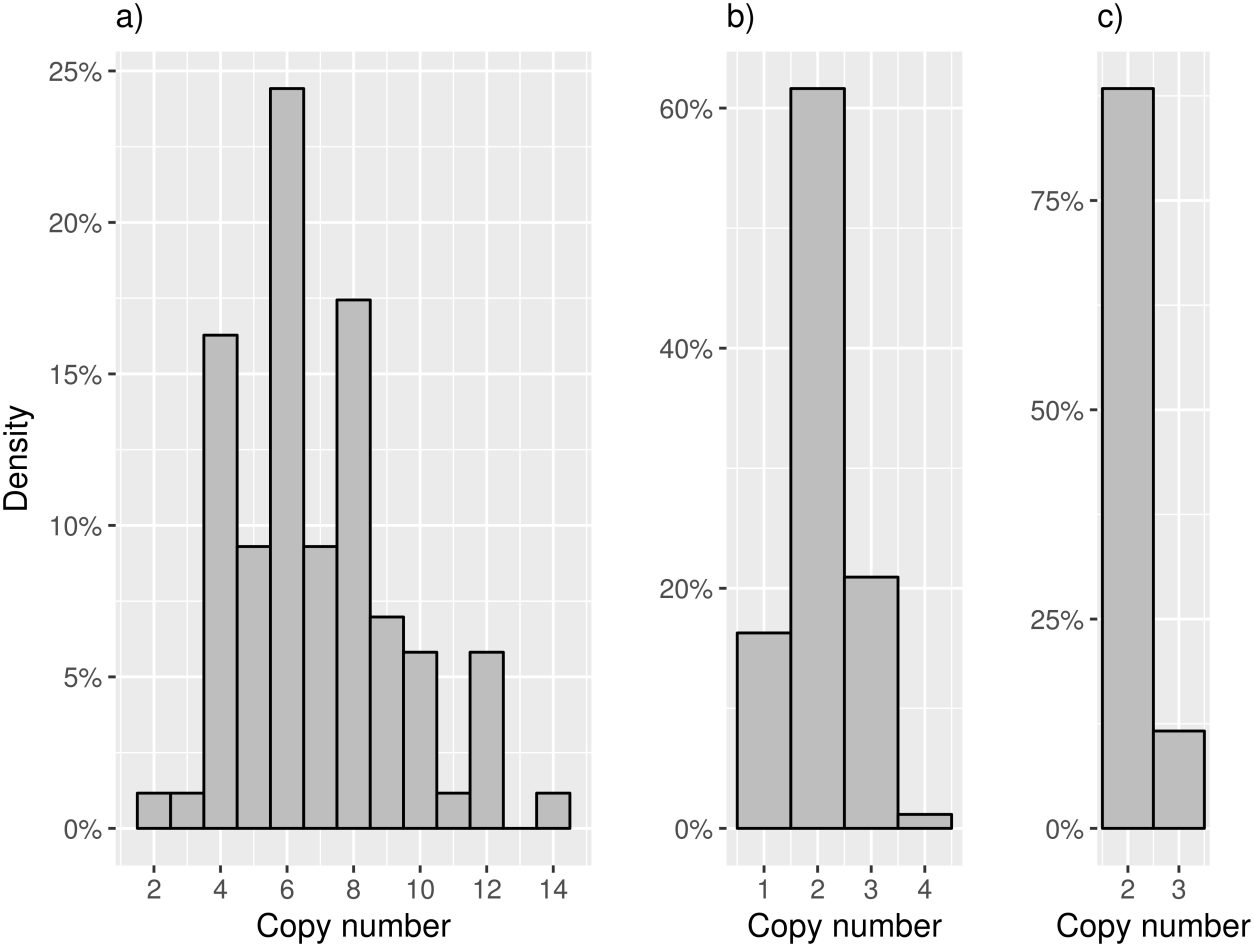
Copy number distributions of the AMY genes. Histograms of the copy number distributions of the three genes in the AMY locus: (a) *AMY1*, (b) *AMY2A* and (c) *AMY2B*.

Lastly, AMYCNE was used to directly estimate the copy number of *AMY1*, *AMY2A* and *AMY2B* in the test cohort (n = 86). The analysis showed that most of the tested individuals (n = 62; 72%) harbored an even number of the *AMY1*, which is similar to the results shown by [15]. *AMY1* was the most copy number variable and *AMY2B* the least variable. It was found that the median copy number of *AMY1*, *AMY2A*, and *AMY2B* was 6, 2 and 2, respectively (Fig 3). Finally, a considerable number of individuals (n = 14) carry a heterozygous deletion of the *AMY2A* gene (Fig 3).

**Fig 4:**
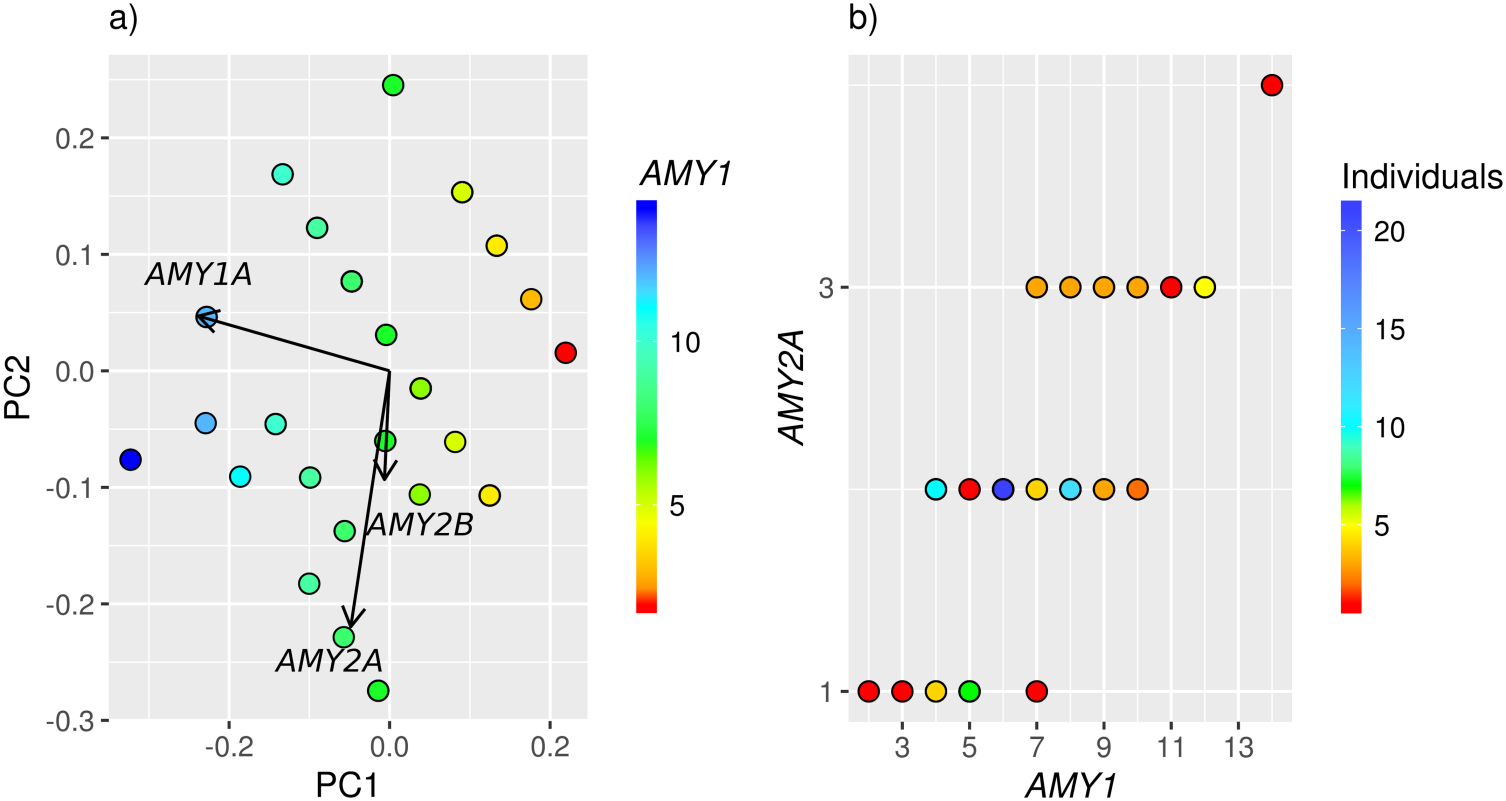
Patterns of the AMY gene copy numbers. (a) A score plot of the copy number distribution of the AMY genes. Each dot is colored according to the *AMY1* copy number of that sample. (b) A scatter plot of the *AMY2A* copy number versus the *AMY1* copy number. The dots are colored according to the number of samples having each combination of *AMY2A* and *AMY1* copy numbers.

Multiple studies have previously suggested interesting relationships between *AMY1* and the *AMY2A* gene [10,15]. Using the AMYCNE copy number predictions, a correlation between the copy numbers of *AMY1* and *AMY2A* was found (Fig 4). These results are similar to other studies of the *AMY* locus within European populations [11,15]. The most common *AMY1*- *AMY2A* copy number pair, is six *AMY1* copies together with two *AMY2A* copies (Fig 4). These are also the two most common copy numbers observed in the cohort (Fig 3).

Previous studies have also shown a high tendency of parity among odd and even copy numbers of *AMY1* and *AMY2A* (i.e if the copy number of *AMY2A* is odd, then the copy number of *AMY1* is likely to be odd as well [15]. In this study, such parity is found only in 64 out of 86 samples (72%), which is considerably lower than the 98% parity reported previously [15]. According to Fig 3, the *AMY1* measurement with AMYCNE should accurately determine the copy number of *AMY1* if it is below 9. To reduce the amount of noise, all samples having a copy number higher than 8 were excluded from the parity analysis. In this sample set, parity was found in 55 out of 68 samples (81%), which is more similar to the results of [15]. Earlier studies using less precise copy number determination methods were not able to find such parity [4,20]. This indicates that AMYCNE is highly precise.

### Copy number estimation of *FCGR3A* and *FCGR3B*

AMYCNE was more accurate than CNVnator predicting the *FCGR3A* copy number (P value = 2.73^−5^) and for *FCGR3B* the two programs produced the exactly same copy numbers (Table 2).

**Table 2:** Comparison of AMYCNE and CNVnator against the truth-set presented in [16].

Copy number estimation of the *FCGR3A* and *FCGR3B* genes						
	*FCGR3A*			*FCGR3B*		
Caller	MAE	SDMAE	Accuracy	MAE	SDMAE	Accuracy
AMYCNE	0.29	0.45	0.71	0.15	0.34	0.85
CNVnator	0.52	0.54	0.5	0.15	0.34	0.85

This was surprising because the copy number distribution for *FCGR3A* and *FCGR3B* in the 164 samples, was very similar. The average copy number for both genes was 2.1 ranging from 1 to 4 copies (standard deviations 0.46 for *FCGR3A* and 0.58 for *FCGR3B*) (S2 Dataset). Hence there are other factors than copy number that cause these differences in performance across the two genes.

### Copy number estimation of mosaic aneuploidies of chromosome X

**Fig 5:**
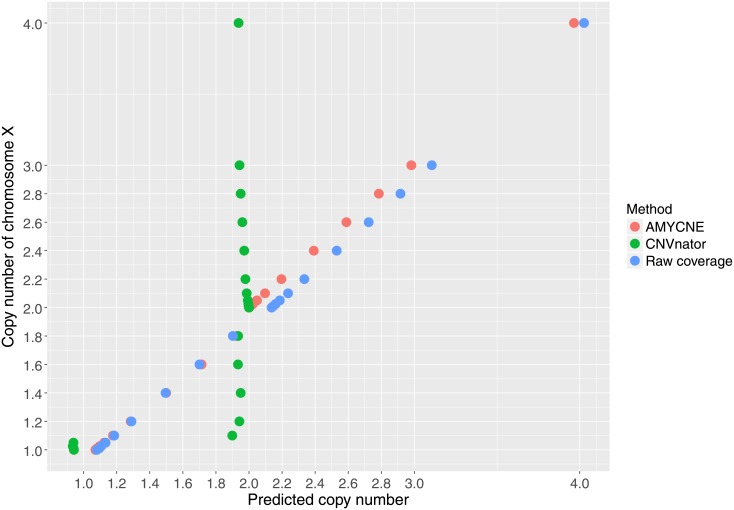
Copy number predictions of mosaic aneuploidies of chromosome X. The predicted X chromosome copy number plotted against the true copy number. The results of the three copy number prediction software are presented in three different colors: red (AMYCNE), green (CNVnator), and blue (Raw coverage estimation).

In the simulated dataset, the comparison between the three different copy number predictions methods (CNVnator, AMYCNE, Raw coverage estimation) showed that AMYCNE was the most accurate method (AMYCNE vs Raw coverage estimation P value = 1.2^−5^; AMYCNE vs CNVnator P value = 3.78^−4^) (Fig 5). CNVnator had the lowest accuracy and predicted that the number of X chromosomes was either one or two for all individuals (Fig 5).

### Annotation of DELLY deletion calls of the hapmap sample NA12878

The structural variant caller DELLY was run on the NA12878 sample, using the DELLY deletion mode. This mode will detect deletions in WGS data using discordant pairs and split read information. The DELLY output VCF file was filtered based on the copy number predicted by AMYCNE. The precision and sensitivity of these calls was assessed using the validated set of variants described in the method section. Using the AMYCNE annotation to filter the DELLY output, the precision is increased (Table 3). The performance of AMYCNE as a filter is highly similar to the PASS filter of the output VCF file. By combining these two filters, the precision of DELLY was further improved.

**Table 3:** Precision and sensitivity of Delly on the NA12878 sample, using various combinations of filters.

Delly deletion calling on NA12878		
Filter	Sensitivity	Precision
No filter	0.92	0.17
AMYCNE filter	0.85	0.56
PASS filter	0.86	0.56
Combined AMYCNE and PASS filter	0.8	0.74

## Discussion

AMYCNE is a highly accurate and precise tool. Benchmarked on the *AMY1* locus, it performs similar to ddPCR (Fig 2), and performs well in a variety of settings (Table 2, Fig 5). Moreover, the results generated by AMYCNE are similar to that of more complex population genomic tools (Fig 4) [20]. AMYCNE is a simple to use tool, requiring only one single individual to estimate the copy number of complex genomic regions. These properties make AMYCNE a useful tool in a clinical environment, where the copy number of all genes in a gene list could be tested automatically based on WGS data, as well as in the research of rare disease genes, where the number of patients in a cohort typically is small and sharing of sensitive patient material is complicated.

A large number of popular structural variant callers do not use read depth for classification or calling of structural variants [13,21]. This leads to a loss of the valuable information found in the read depth of the sequencing data. The annotation mode of AMYCNE allows for the integration of read depth data for these kinds of structural variant callers, which can be used to increase the precision of such callers (Table 3).

Using AMYCNE to study the copy number distributions of the *AMY* genes, we find that the *AMY* copy number distribution of the Swedish population is similar to that of other European populations [15]. As is similar to earlier studies, we found an interesting relationship in the copy number of the *AMY* genes, where AMYCNE detects a positive correlation between *AMY1* and *AMY2A* (Fig 3). However, the parity of the *AMY2A* and *AMY1* is less clear than shown previously [15].

To conclude, AMYCNE is an accurate tool for estimating high copy numbers in complex genomic regions using WGS data. The tool was validated using a variety of data sources and loci. Finally, AMYCNE was used to characterize the complex *AMY1*-*AMY2* region in our cohort. In aggregate, the data shows that AMYCNE could be used to complement WGS structural variant callers.

## Supporting information

S1 Fig The AMY loci. A diagram of the *AMY1* loci.
(PNG)

S1 Text Supplementary methods. (DOCX)

S1 Dataset *AMY1* copy number measurements. Measurements of the *AMY1* copy number, using ddPCR, AMYCNE, CNVnator, and Diploid copy number estimation of *AMY1*.
(ZIP)

S2 Dataset *FCGR3A* and *FCGR3B* copy number measurements. Measurements of the copy number of *FCGR3A* and *FCGR3B*, using AMYCNE and CNVnator.
(ZIP)

S1 Table Genotyping simulated mosaic aneuploidies of chromosome X. Measurements of the copy number of chromosome X, using AMYCNE, CNVnator and estimations based on raw coverage data.
(XLSX)
