# Supplementary material for: AMYCNE: Confident copy number assessment using whole genome sequencing data
Source: PLoS One. 2018 Mar 26;13(3):e0189710. doi: 10.1371/journal.pone.0189710 (PMC5868770; doi:10.1371/journal.pone.0189710)
Supplement: S1 Text — (DOCX) [file pone.0189710.s002.docx]

**AMYCNE: Confident copy number assessment using whole genome sequencing**

Jesper Eisfeldt1,2,*, Daniel Nilsson1,2,4, Johanna C. Andersson-Assarsson3 and Anna Lindstrand1,4

1Department of Molecular Medicine and Surgery, and Center for Molecular Medicine, Karolinska Institutet, 171 76 Stockholm, Sweden

2Science for Life Laboratory, Karolinska Institutet Science Park, 171 21 Solna, Sweden

3Department of Molecular and Clinical Medicine, Sahlgrenska Academy, University of Gothenburg, 413 45 Gothenburg, Sweden

4Department of Clinical Genetics, Karolinska University Hospital, 171 76 Stockholm, Sweden

*Corresponding author

Email: [jesper.eisfeldt@scilifelab.se](mailto:jesper.eisfeldt@scilifelab.se) (KI)

**Generate coverage bed files using TIDDIT**

BED files containing the binned coverage across the entire genome was generated using TIDDIT-1.0.

TIDDIT may be downloaded via the following link:

*https://github.com/SciLifeLab/TIDDIT/releases*

The BED files were generated through the following command:

*./TIDDIT --cov -b INPUT_BAM -o OUPUT_PREFIX –bin_size 100*

where INPUT_BAM is the input bam file and OUTPUT_PREFIX is the output prefix of the bed file. The BED files are then used as the input of AMYCNE.

***AMY1* copy number estimation using AMYCNE**

The following command was used to estimate the copy number the *AMY1* gene:

*python AMYCNE.py --genotype --gc GC_tab_file.tab --coverage INPUT_BED --region AMY1.txt --Q 0*

where INPUT_BED is the coverage bed file generated by TIDDIT. The quality threshold (Q) is set to 0, since it is not possible to map the reads of the AMY1 gene uniquely (the reads are spread across *AMY1A*, *AMY1B*, and *AMY1C*) [1].

The AMY1.txt file containing the positions of *AMY1A*, *AMY1B*, and *AMY1C* may be downloaded through the AMYCNE repository:

*https://github.com/J35P312/AMYCNE/tree/master/benchmarking_and_examples/AMY_genotyping*

The GC_tab_file.tab is the BED file describing the GC-content in bins of size 100 bases across the entire genome. This file may be downloaded through the AMYCNE repository as well:

*https://github.com/J35P312/AMYCNE/tree/master/benchmarking_and_examples/delly_filtering/GC_tab*

***AMY1* copy number estimation using diploid copy number estimation of *AMY1***

The diploid copy number estimation (DCNE) of AMY1 method utilize a region known as region Z to normalise the coverage across *AMY1A*, *AMY1B*, and *AMY1C* [1]. This region is located on chromosome 1:104 400 000–104 450 000 and is known to be well conserved [1]. The copy number of *AMY1* was found by computing the copy number across region *Z*. Lastly, the coverage across *AMY1A*, *AMY1B*, and *AMY1C* was summed, multiplied by two, and divided by the coverage across Z. The resulting number was rounded and reported as the copy number of *AMY1*.

This algorithm was implemented as a python script. This script is available through the AMYCNE repositor, and is run using the following settings:

*python Diploid_copy_estimation_AMY1.py –coverage INPUT_BED –region AMY1.txt –ref_region Z.txt*

Here, INPUT_BED is the input coverage bed file, AMY1.txt is a BED file giving the position of *AMY1A*, *AMY1B*, and *AMY1C*, while Z.txt is a BED file containing the position of region Z.

***AMY1* copy number estimation using CNVnator 0.3.2**

cnvnator 0.3.2 [2] was used as a third method of genotyping *AMY1*. Initially, variant calling was performed using bins of size 1kb:

*cnvnator -root $2.root -tree $1*

*cnvnator -root $2.root -his 1000 -d reference_dir*

*cnvnator -root $2.root -stat 1000 >> $2.cnvnator.log*

*cnvnator -root $2.root -partition 1000*

*cnvnator -root $2.root -call 1000 > $2.out*

*cnvnator2VCF.pl $2.out > $2.cnvnator.vcf*

Here, $2 is the output prefix, and $1 is the input bam. Upon finishing variant calling, the output VCF of each sample was analysed using the AMY1_cnvnator_measurement.py script:

*python AMY1_cnvnator_measurement.py INPUT_VCF*

where INPUT_VCF is the vcf generated by cnvnator. This script uses the cnvnator normalized read depth, which is reported as an entry in the VCF info-field to estimate the copy number of *AMY1*.

The script searches for all called copy number variants covering any of the *AMY1* genes. Thereafter, for each *AMY1* gene, the normalised read depth of each variant is added to the normalised read depth of that *AMY1* gene, such that:

$$Normalised Read depth of AMY1=1+\sum_{i=0}^{n_{AMY1}} {Ovar}_{i}^{AMY1}*{(RDvar}_{i}^{AMY1}-1)$$

Here, $n_{AMY1}$ is the number of detected variants within that *AMY1* gene, ${Ovar}_{i}^{AMY1}$ is the fraction of bases of the *AMY1* gene that is covered by variant ${var}_{i}^{AMY1}$, and lastly, ${RDvar}_{i}^{AMY1}$ is the normalised read depth of ${var}_{i}^{AMY1}$. Once this formula is computed for *AMY1A*, *AMY1B*, and *AMY1C*, their normalised read depth is added and multiplied by two *i.e.* the ploidy of the human genome.

While CNVnator does have a genotyping module, this module reports only the estimated copy number across one given region. Since *AMY1A*, *AMY1B*, and *AMY1C* are highly similar (99% similarity [1]), the reads will be equally spread between these genes. Hence, predicting the copy number across one *AMY1* gene at a time will lead to round-off errors. Therefore, the previously described custom genotyping method was implemented to assess the copy number of *AMY1* using CNVnator.

**Wilcoxon signed rank sum test and comparisons to ddPCR**

The Wilcoxon signed ranks sum test was used to test if the prediction of AMYCNE was significantly closer to the ddPCR measurements compared to those of CNVnator or DCNE of *AMY1*. This test was performed by creating a list for each one of the three methods. The value of each entry in each list was the absolute value of the difference between the ddPCR measurement and the prediction of one of the whole genome sequencing prediction methods. Thereafter, the Wilcoxon rank sum test was employed to compare the list of AMYCNE to the list of CNVnator and DCNE of *AMY1*, individually.

**Retrieval of thousand genome samples**

The Thousand genomes low coverage data [3] was used to benchmark AMYCNE on the *FCGR3A* and *FCGR3B* genes. These low coverage bam files were retrieved via UPPMAX (<http://www.uppmax.uu.se/resources/databases/1000-genomes-project/>). In total, 164 individuals were analysed; these were all the available samples that had been genotyped through the study [4].

**Estimation of the *FCGR3A* and *FCGR3B* copy numbers using AMYCNE**

The following command was used to estimate the copy number the *FCGR3A* and *FCGR3B* genes:

*python AMYCNE.py --genotype --gc GC_tab_file.tab --coverage INPUT_BED –region FCGR3.txt*

where INPUT_BED is the coverage bed file generated by TIDDIT. The maximum coverage threshold is set to 25; bins having higher coverage than this value are excluded from the analysis. The FCGR.txt file is a text file containing these two lines:

*sum(1:161592988-161601753)*

*sum(1:161511551-161520413)*

These two lines provide the coordinates of the genomic positions of *FCGR3A* and *FCGR3B* in the hg19 reference genome. The GC_tab_file.tab is the BED file describing the GC-content in bins of size 100 bases across the entire genome. This file may be downloaded through the AMYCNE repository as well:

*https://github.com/J35P312/AMYCNE/tree/master/benchmarking_and_examples/delly_filtering/GC_tab*

**Estimation of the *FCGR3A* and *FCGR3B* copy numbers using CNVnator 0.3.2**

cnvnator 0.3.2 [2] was used as a second method for copy number estimation of the *FCGR3A* and *FCGR3B* genes. Initially, CNVnator root files were prepared for each sample, using the following commands:

*cnvnator -root $2.root -tree $1*

*cnvnator -root $2.root -his 500 -d reference_dir*

*cnvnator -root $2.root -stat 500 >> $2.cnvnator.log*

*cnvnator -root $2.root -partition 500*

Here, $2 is the output prefix, and $1 is the input bam. The 500 base pair bin size was chosen according to the recommendations of the CNVnator manual. Once the root files were ready, the *FCGR3A* and *FCGR3B* genes were genotyped using the following command:

*cnvnator -root $1 -genotype 500 << EOF*

*1:161592988-161601753*

*1:161511551-161520413*

*exit*

*EOF*

Here, $1 is the root file generated through the previously described command.

**Simulation of mosaic aneuploidy of chromosome X**

The Illumina WGS data of 20 female individuals carrying various grades of mosaic aneuploidies of chromosome X were simulated using Simseq (https://github.com/jstjohn/SimSeq). The sequencing data of these individuals was generated so that the average coverage became 20X.

Additionally, 8000 deletions and 8000 duplications, sized 1- 150 000 base-pairs were added to each of these simulated genomes. These CNVs were added to represent the normal variation of the human genome.

The simulated genomes carry mosaic monosomies and trisomies (present in 1%, 2.5%, 5%,10%,20%, 40%, 60%, 80%, and 100%), as well as tetrasomy of chromosome X. Additionally; the genome of an individual carrying a normal karyotype was generated.

The pipeline as well as instructions on how to run the WGS data simulation pipeline is available through the following repository:

*https://github.com/J35P312/aneuploidyX*

The genomes were created by generating 10 bam files (coverage 0.1X, 0.25X, 0.5X, 1X, 2X, 4X, 6X, 8X, 10X, and 20X). The trisomy cases were generated by adding chromosome X of each low coverage bam file to the 20X bam file; emulating 9 samples carrying various degrees of mosaic trisomy X. These operations were performed using samtools; first chromosome X was extracted from the low coverage bam files using samtools view, resulting in 9 bam files containing only chromome X. Thereafter each of these bam files were merged with the 20X bam file using samtools merge; resulting in 9 samples carrying various degrees of mosaic trisomy X.

Instead, the monosomy samples were generated by adding chromosome X of each bam file having lower coverage than 10X to the 10X coverage bam file. Thereafter, chromosome X was removed from the 20X bam file. Lastly, each one of the previously described bam files were added to the 20X bam file lacking chromosome X; resulting in various degrees of mosaic monosomy of chromosome X. Similar to the trisomy samples, these operations were performed through series of applying samtools view and samtools merge.

Lastly, the tetrasomy sample was created by extracting chromosome X from the 10X coverage bam file, and adding two copies of the resulting 10X chromosome X bam file to the 20X bam file.

**Chromosome X copy number estimation using AMYCNE**

The following command was used to estimate the copy number of chromosome X:

*python AMYCNE.py --genotype --gc GC_tab_file.tab --coverage INPUT_BED –X.txt*

Where INPUT_BED is the coverage bed file generated by TIDDIT, and the X.txt file is a text file containing the following line:

*sum(X:1-155270560)*

Which provides the entire length of chromosome X. The GC_tab_file.tab is the BED file describing the GC-content in bins of size 100 bases across the entire genome. This file may be downloaded through the AMYCNE repository:

*https://github.com/J35P312/AMYCNE/tree/master/benchmarking_and_examples/delly_filtering/GC_tab*

**Chromosome X copy number estimation using CNVnator 0.3.2**

cnvnator 0.3.2 [2] was used as a second method for copy number estimation of chromosome X of the simulated aneuploidy samples. CNVnator root files were prepared for each sample, using the following commands:

*cnvnator -root $2.root -tree $1*

*cnvnator -root $2.root -his 100 -d reference_dir*

*cnvnator -root $2.root -stat 100 >> $2.cnvnator.log*

*cnvnator -root $2.root -partition 100*

Here, $2 is the output prefix, and $1 is the input bam. Once the root files were ready, the copy number of chromosome X was estimated through the following command:

*cnvnator -root $1 -genotype 100 << EOF*

*X:1-155270560*

*exit*

*EOF*

Here, $1 is the root file generated through the previously described command.

**Chromosome X copy number estimation using raw coverage data**

Additionally, the copy number of chromosome X was estimated based on the raw coverage. The raw coverage estimation was performed by computing the average coverage across chromosome X, as well as the entire genomic coverage. Thereafter the ratio of these two numbers were computed:

*Coverage_X_/Coverage_genome_*

The copy number of chromosome X was obtained by multiplying that ratio by two. The coverage data was obtained from the same TIDDIT bed files as those used by AMYCNE, and the script used for these computations is available through the repository:

*https://github.com/J35P312/aneuploidyX*

**Downloading of NA12878**

The downsampled NA12878 BAM file was downloaded via the link:

*ftp://ftp-trace.ncbi.nlm.nih.gov/giab/ftp/data/NA12878/NIST_NA12878_HG001_HiSeq_300x/RMNISTHS_30xdownsample.bam*

More information about this BAM file may be found in the following document:

*ftp://ftp-trace.ncbi.nlm.nih.gov/giab/ftp/data/NA12878/NIST_NA12878_HG001_HiSeq_300x/README_NIST_Illumina_pairedend_NA12878.txt*

Additional information may also be found in the preprint online [5].

**SV calling on NA12878 using Delly 0.7.2**

Delly 0.7.2 [6] was used to call SV on the NA12878 sample using command:

*delly -t DEL -o NA12878.DEL.vcf -g human_g1k_v37.fasta RMNISTHS_30xdownsample.bam*

Delly may be downloaded through the git hub repository:

*https://github.com/dellytools/delly/releases*

**AMYCNE copy number annotation module**

The AMYCNE annotation module is used to add copy number annotation to the variants of a structural variant VCF file, produced by callers such as Delly. The copy number of delly calls was annotated

using the following command:

*python AMYCNE.py --annotate --gc GC_tab_file.tab --coverage INPUT_BED --region AMY1.txt –vcf NA12878.DEL.vcf > NA12878.DEL.annotated.vcf*

**AMYCNE deletion filter and benchmarking**

The purpose of the AMYCNE filter script is to present a use case for the AMYCNE annotation module, as well as to validate the accuracy of AMYCNE. The AMYCNE deletion filter is a python script developed to remove false deletion calls based on the AMYCNE annotation. The user of this script should be careful when using this filter, since it may remove mislabelled variants, or low quality deletions of importance. In a more realistic scenario, multiple factors, such as the caller statistics i.e. read pairs or split reads, as well additional information, such as gene or frequency annotation, should be taken in account.

The filter script utilizes three statistics provided by AMYCNE to determine the quality of a variant. These three statistics are the predicted copy number, the 95% confidence interval around the copy number, as well as the fractions of bins that passed the quality tests.

The filter script combines these three statistics into two tests. If both tests are passed, the deletion is printed to a high quality deletion file, otherwise it is discarded.

In the first test, the fraction of low quality bins is utilized to filter the deletions. The variant will pass this quality check if the majority of the bins within the same region as the variant is of high quality. The second test combines the predicted copy number and the confidence interval. A variant will pass this test if the predicted copy number is less than two, or if the lower bound of the interval is less than 1.5 copies. In a female individual, any deletion on the Y chromosome is considered low quality.

The filter is found in the AMYCNE git hub repository:

*https://github.com/J35P312/AMYCNE/*

and is run through the following command:

*python AMYCNE_deletion_filter.py > filtered_vcf.vcf*

The same command was used to filter the Delly deletion call of the NA12878 sample:

*python AMYCNE_deletion_filter.py NA12878.DEL.annotated.vcf > NA12878.DEL.filt.vcf*

Lastly, the precision and sensitivity of these four settings were tested using a set of validated deletions, available through the supplementary materials of the Svclassify manuscript [7]:

*ftp://ftp-trace.ncbi.nlm.nih.gov/giab/ftp/technical/svclassify_Manuscript/Supplementary_Information/Personalis_1000_Genomes_deduplicated_deletions.bed*

A called variant and a variant of the truth set was considered to be the same if their reciprocal overlap exceeded 0.6. The reciprocal overlap, as well as sensitivity and precision, was computed using the

benchmarking_NA.sh script found in the AMYCNE repository:

*https://github.com/J35P312/AMYCNE/blob/master/benchmarking_and_examples/delly_filtering/benchmarking_NA.sh*

Prior to analysing*,* the sensitivity and precision of Delly, all variants smaller than 500 base pairs were removed from the truth set, as well as the Delly VCF files. The reason for this was that AMYCNE is designed to analyse large CNVS. Moreover, the truth set was converted to a BEDPE file having the following format:

*ChrA ChrB StartA EndA StartB EndB*

The resulting truth set is available through the AMYCNE repository:

*https://github.com/J35P312/AMYCNE/tree/master/benchmarking_and_examples/delly_filtering/NA12878_500.db*

Finally, the following two commands were issued to compute the precision of the original delly VCF file, as well as the AMYCNE annotated and filtered file:

./benchmarking_NA.sh *NA12878.DEL.vcf*

./benchmarking_NA.sh *NA12878.DEL.filt.vcf*

The benchmarking_NA.sh script reports the sensitivity and precision of the input VCF, as well as the quality filtered input vcf. This filter quality filter removes any call whose quality flag is not set to pass, PASS, or “.”.

**References**

1 Rare variant discovery by deep whole-genome sequencing of 1,070 japanese individuals. Nature communications 2015; 6:8018 doi: 10.1038/ncomms9018.

2 Abyzov, Alexej, et al. "CNVnator: an approach to discover, genotype, and characterize typical and atypical CNVs from family and population genome sequencing." *Genome research* 21.6 (2011): 974-984.

3 1000 Genomes Project Consortium. A global reference for human genetic variation. Nature. 2015 Oct 1;526(7571):68.

4 Qi YY, Zhou XJ, Bu DF, Hou P, Lv JC, Zhang H. Comparison of Multiple Methods for Determination of *FCGR3A/B* Genomic Copy Numbers in HapMap Asian Populations with Two Public Databases. Frontiers in genetics. 2016;7.

5 Eberle, Michael A., et al. "A reference data set of 5.4 million phased human variants validated by genetic inheritance from sequencing a three-generation 17-member pedigree." *Genome research* 27.1 (2017): 157-164.

6 Rausch, Tobias, et al. "DELLY: structural variant discovery by integrated paired-end and split-read analysis." *Bioinformatics* 28.18 (2012): i333-i339.

7 Parikh, Hemang, et al. "svclassify: a method to establish benchmark structural variant calls." *BMC genomics* 17.1 (2016): 64.
